# Effect of Exercise on Brain Health: The Potential Role of Lactate as a Myokine

**DOI:** 10.3390/metabo11120813

**Published:** 2021-11-29

**Authors:** Takeshi Hashimoto, Hayato Tsukamoto, Soichi Ando, Shigehiko Ogoh

**Affiliations:** 1 Faculty of Sport and Health Science, Ritsumeikan University, Shiga 525-8577, Japan; thashimo@fc.ritsumei.ac.jp (T.H.); h-tsuka@fc.ritsumei.ac.jp (H.T.); 2 Graduate School of Informatics and Engineering, The University of Electro-Communications, Tokyo 182-8585, Japan; soichi.ando@uec.ac.jp; 3 Department of Biomedical Engineering, Toyo University, Saitama 350-8585, Japan

**Keywords:** executive function, mental health, brain-derived neurotrophic factor, insulin-like growth factor-1, vascular endothelial growth factor, neurogenesis, angiogenesis, cerebral blood flow, nicotinamide adenine dinucleotide hydrate

## Abstract

It has been well established in epidemiological studies and randomized controlled trials that habitual exercise is beneficial for brain health, such as cognition and mental health. Generally, it may be reasonable to say that the physiological benefits of acute exercise can prevent brain disorders in late life if such exercise is habitually/chronically conducted. However, the mechanisms of improvement in brain function via chronic exercise remain incompletely understood because such mechanisms are assumed to be multifactorial, such as the adaptation of repeated acute exercise. This review postulates that cerebral metabolism may be an important physiological factor that determines brain function. Among metabolites, the provision of lactate to meet elevated neural activity and regulate the cerebrovascular system and redox states in response to exercise may be responsible for exercise-enhanced brain health. Here, we summarize the current knowledge regarding the influence of exercise on brain health, particularly cognitive performance, with the underlying mechanisms by means of lactate. Regarding the influence of chronic exercise on brain function, the relevance of exercise intensity and modality, particularly high-intensity interval exercise, is acknowledged to induce “metabolic myokine” (i.e., lactate) for brain health.

## 1. Introduction

It has been well established that habitual exercise is beneficial for the cognition and brain health of most individuals, including older adults [1,2]. This view is not surprising because it is said that “exercise is the real polypill” based on organ-induced peripheral factors [3]. In general, it has been considered that the effects of habitual exercise on the human body are the result of repeated exercise and thus may be associated with cumulative acute responses to exercise.

Similarly, it may be reasonable to say that acute exercise favorable for improving brain function, although this is a transient response, is also beneficial for brain health with continuous repetition via chronic exercise training. However, the mechanisms of chronic exercise-improved brain function, especially how the effect of acute exercise on brain function determines that of chronic exercise, remain incompletely unknown. For instance, chronic exercise effects can be modified using the same acute exercise by changing exercise strength, duration, and frequency. Hence, the proper exercise prescription for chronic brain health may be difficult to build from results on the effect of acute exercise on brain function. Nonetheless, it is important to explore and organize the underlying mechanisms of acute exercise for brain health to provide insight into proper exercise prescriptions.

Among the acute responses to exercise, a growing body of evidence is accumulating to suggest that the myokine (i.e., muscle-induced peripheral factors) cathepsin B and irisin pass through the blood–brain barrier to enhance brain-derived neurotrophic factor (BDNF) production and hence improve neurogenesis, memory, and learning [4]. On the other hand, lactate, as an exercise-induced myokine favorable to the brain, was not investigated to identify the mechanism of exercise-induced improvement in brain function, although the production of lactate has been widely used as a biomarker to reflect exercise mode, strength, and duration [5,6,7,8].

In this minireview, we summarized the possibility of lactate as one of the underlying mechanisms linking brain health outcomes, particularly cognitive performance and mental health, to exercise regimens.

## 2. Exercise Intensity and Modality for Brain Health Regarding Chronic Exercise Adaptation (Implication of Lactate)

**Figure 1:**
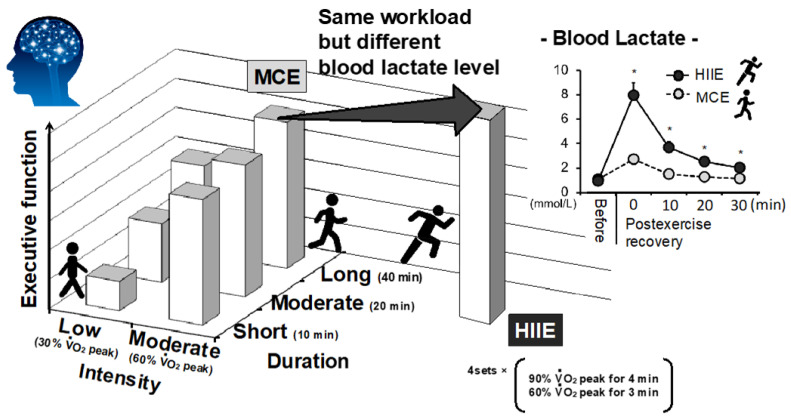
Impact of exercise intensity, duration, and modality on acute enhancement of executive function. The graph is illustrated by the authors based on previous studies [7,8,18]. HIIE could improve EF rather than volume-matched (i.e., same workload) MCE with more lactate production during postexercise recovery period [7]. * *p* < 0.05 vs. MCE.

To promote and maintain health, the American College of Sports Medicine (ACSM) and American Heart Association (AHA) recommends that healthy adults aged 18–65 years perform sufficient volumes of exercise, such as moderate-intensity exercise for at least 30 min for 5 days/week or vigorous-intensity exercise for 20 min for 3 days/week [9]. Importantly, compared to habitual lower-intensity exercise, higher-intensity exercise can effectively improve cardiovascular and metabolic health [10,11,12]. In particular, long-term/chronic high-intensity interval exercise (HIIE) training (i.e., HIIT) is more effective than long-term/chronic moderate-intensity continuous exercise (MCE) because it increases exercise capacity in addition to cardiovascular and metabolic health in healthy individuals [13,14,15]. The effectiveness of HIIT over MCE training is also relevant for brain health. Recently, Mekari et al. demonstrated that HIIT was more effective for the improvement of executive function (EF) than MCE training in young adults [16]. A recent meta-analysis indicated that HIIT might be more effective for improving severe mental illness (e.g., cognition, negative and positive symptoms of schizophrenia, and depressive mood) than moderate-intensity exercise [17]. Given that HIIE produces more lactate than general exercise modalities, such as MCE, some beneficial effects of lactate on health, including brain health, can be implicated. For instance, based on the notion that acute exercise that is favorable for improving brain function is also beneficial for brain health with continuous repetition via chronic exercise training, our previous study demonstrated that HIIE could improve EF rather than MCE and was accompanied by more lactate production (Figure 1) [7], which may imply a potential benefit of lactate on increased cognitive performance by HIIE and subsequent HIIT.

## 3. Chronic Cognitive and Mental Alterations with Regular Exercise and Its Potential Link to Chronic Exercise-Induced Anatomical and Cerebral Microvasculature Alterations

The potential mechanisms of habitual exercise/physical activity-induced improvement as well as aging-induced impairments in cognitive performance and mental health remain unclear but are assumed to be associated with several physiological factors. For instance, the deleterious effects of aging on the brain comprise negative physiological and anatomical alterations, e.g., hemodynamic activity, synaptic plasticity, decreased brain volume and neurogenesis, while physical activity prevents the deleterious effects on the brain and, in contrast, induces brain neural alterations, including the formation of new neurons, the proliferation of neural cells, and integrated functional neural networks [19,20]. In particular, structural alterations, such as increased neurogenesis, synaptogenesis, angiogenesis, and brain volume, seem to be characteristics of the beneficial effects of chronic exercise on cognitive performance and mental health [2].

Regular aerobic exercise can increase or preserve the regional brain volume in areas associated with cognitive decline and portions of mental health [21,22,23]. It has been reported that aerobic exercise (i.e., 6 to 12 months of a walking program) increases spatial memory as well as gray and white matter volumes in both temporal (including the hippocampi) and prefrontal regions in healthy older adults (without dementia) [24]. In addition, Jonasson et al. demonstrated that following a 6-month exercise training period, the change in “cognitive score” determined by episodic memory, updating, processing speed, and EF was positively related to the thickness of the dorsolateral prefrontal cortex [25]. Regarding mental health, patients with major depressive disorder or schizophrenia show decreased hippocampal or gray matter volume [26,27], while an exercise-induced increase in hippocampal volume can be related to cognitive performance even in patients with schizophrenia [22]. However, whether brain structure is associated with psychiatric and neurological disorders is controversial [28], and whether the positive effects of aerobic exercise can be extended to psychiatric disorders is still unclear [21]. Further studies are needed to uncover the pathophysiology of mental disorders and improve the effect of exercise or physical activity.

In addition to brain structural/anatomical alterations, changes in cerebral microvasculature function can be a physiological factor that may elicit exercise-enhanced brain function. Since the energy reserve of the brain is relatively small, a continuous supply of glucose and oxygen from the cerebral circulation to the brain is required to maintain its function, e.g., cognitive performance. Thus, especially in the brain, synaptic activity suddenly increases the demand for energy for maintaining brain function and consequently might cause a relative lack of oxygen and glucose. However, in the brain, the neural activity causes neurovascular coupling with accordingly transient and adequate increases in regional cerebral blood flow (CBF) and consequently partially maintains brain function [29]. Indeed, the onset of cognitive impairment often occurs following cerebrovascular dysfunction, suggesting that dysfunction of CBF regulation is one of the mechanisms of the onset of dementia [30]. Furthermore, a decrease in the response of regional CBF to a simple motor task occurs when either intracranial carotid arteries or one vertebral artery is occluded in asymptomatic patients [31]. In addition, neural coupling to several physiological stimuli and resting CBF are reduced in patients with Alzheimer’s disease [32,33,34,35,36]. These findings indicate that brain function via neurovascular coupling is attenuated by inadequate global or focal CBF regulation; thus, the regulation of global CBF is important to maintain adequate neural coupling [29] and thus brain function.

## 4. Can Acute Alterations in CBF to Exercise Affect Cognitive Performance?

As mentioned above, it is expected that maintaining brain function requires adequate CBF regulation as an important physiological factor. However, no study has examined whether alterations in CBF directly modify cognitive performance because CBF cannot be isolated from the many physiological factors that affect cognitive performance in patients with cerebral disease, vascular disease, or dementia, as well as in healthy older adults.

Basically, augmented cerebral metabolism or cerebral neural activity [37,38,39] are accompanied by transient increases in CBF [40,41,42] as well as cognitive performance [43,44] during and/or following mild- to moderate-intensity aerobic exercise. In contrast, similar to the decrease in CBF associated with hyperventilation during prolonged or heavy aerobic exercise [41], the exercise-induced facilitation of cognitive performance disappears during such prolonged exercise [45]. From this background, we previously examined for the first time whether manipulation of CBF alteration affects cognitive performance in young, healthy participants [46]. In contrast to our hypothesis, however, cognitive performance improved in response to the decrease in CBF during prolonged heavy exercise, and unexpectedly, an isolated change (i.e., hypercapnia-induced increase) in CBF did not affect cognitive performance at rest or during exercise [46]. Furthermore, several studies reported that increases in CBF during exercise were not directly related to changes in cognitive performance [47,48]. These findings suggest that acute exercise-induced cognitive improvement may not have the same narrative as that of chronic exercise in terms of the cerebrovascular system; thus, it is not simply due to an increase in global CBF, implying that another factor modified by exercise, rather than a change in CBF, affects cognitive performance.

## 5. Cerebral Lactate Metabolism and Cognitive Performance

A decrease in cerebral oxygenation is induced by prolonged exercise [46,49] or exercise under mild or severe hypoxia [50,51], while impaired cognitive performance is not evident in healthy young participants, suggesting a dissociation between an alteration in CBF and subsequent change in oxygen delivery to the brain and cerebral metabolism or cognitive performance. Indeed, albeit with a reduction in CBF during heavy exercise, the elevation of brain neural activity and metabolism might be accompanied by compensatory increases in the uptake of lactate, glucose, and oxygen support for the brain (arterial-jugular venous difference) [37]. Given that augmented brain neural activity and metabolism are independent of increases in CBF [52], extensive activation of motor and sensory systems due to the higher-order function of the prefrontal cortex may affect cognitive performance rather than cerebral perfusion in response to exercise.

**Figure 2:**
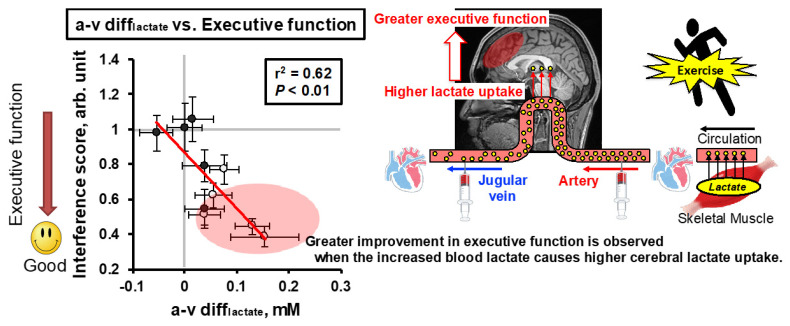
Relationship between a-v diff_lactate_ and Δinterference score (i.e., executive function) during postexercise recovery. The open circles indicate the average of each time point during the post-first bout of HIIE recovery, and the solid circles indicate the average of each time point during the post-second bout of HIIE recovery in which a lower systemic lactate concentration is observed. This result suggests that brain lactate uptake is associated with better executive function. Values are the means ± SEM. Modified/adopted from Hashimoto et al. [67].

Regarding metabolism, although the brain relies mainly on glucose at rest, during high-intensity exercise, the brain becomes dependent on lactate delivery [53,54] and repeated HIIE, which attenuates the increase in systemic blood lactate, resulting in impaired maintenance of HIIE-enhanced cognitive performance (i.e., EF) [18]. In particular, HIIE may facilitate neuronal activation and excitation levels to the extent that summation is facilitated to improve cognitive performance [7,55,56]. Neuronal activation is associated with an increase in energy requirements due to the transport of neurotransmitters and ions [57], and neurons preferentially utilize lactate as a fuel in vivo [58]. Sustained elevation of arterial/systemic lactate in response to intense exercise promotes the supply of lactate as an energy substrate to meet acute neuronal energy requirements [59,60,61]. In addition, intravenous infusion of 100 mM L-lactate into rats promoted cognitive recovery by preserving cerebral ATP generation following traumatic brain injury [62]. Furthermore, Skriver et al. found a correlation between systemic lactate concentration and the acquisition and retention of motor skills [63]. In addition, lactate supports synaptic activity [64], long-term potentiation and memory formation [65], and neuronal plasticity [66]. These findings suggest that brain function as expressed by cognitive performance depends on the provision of lactate. Indeed, we manipulated blood lactate during exercise at a given intensity by repeated HIIE and evaluated whether such manipulation of peripheral lactate metabolism affects brain lactate uptake (i.e., the arterial–jugular venous difference in lactate (a-v diff_lactate_)) and EF [67]. We found that brain lactate uptake is associated with the arterial lactate concentration, and inadequate lactate provision to the brain might attenuate exercise (i.e., HIIE)-enhanced EF [67], irrespective of increased BDNF and catecholamine, both of which are supposed to relate to cognitive performance [56,68,69] (Figure 2). Given the reliance on lactate as a fuel for the brain, variations in blood lactate could affect cognitive performance during and after exercise and account for the significance of exercise (i.e., muscle contraction) for brain function.

On the other hand, a recent study demonstrated that chronic lactate administration to mice promotes hippocampal neurogenesis but does not affect cognitive performance [70]. In addition, Sudo et al. found that recovery of prefrontal oxygenation affected cognitive performance after exhaustive exercise, irrespective of the blood lactate concentration [71]. Further studies are warranted to understand the role of lactate in brain function in acute and chronic exercise.

## 6. Exercise-Induced Improvement in Brain Health Based on Chronic Anatomical and Cerebral Microvasculature Alterations and Its Potential Link to Exercise-Produced Lactate in Active Muscle

As described above, brain structure may determine CBF regulation and volume that affects brain function. Of note, physical activity is useful to upregulate neurotrophins and growth factors, such as BDNF, insulin-like growth factor-1 (IGF-1), and vascular endothelial growth factor (VEGF), which are necessary to maintain existing neurons and neurogenesis for continued brain development [20]. The increases in BDNF, VEGF, and IGF-1 levels are positively related to augmented hippocampal volume, neurogenesis, and angiogenesis, thereby increasing cognitive performance, such as spatial memory, in older adults [20,21,24,72].

Among the growth factors, BDNF might be a key factor involved in cognitive performance improvement, at least regarding memory function and mental health, by means of promoting neurogenesis, synaptic plasticity, and cell survival, particularly in the cerebral cortex and hippocampus [21,73]. Indeed, poor cognitive function and mental health are associated with low circulating BDNF levels in both young and elderly persons and patients with a major depressive disorder [69,74,75]. On the other hand, Griffin et al. (2011) suggested that postexercise improvement in short-term memory performance was related to an acute increase in BDNF [69]. Additionally, to maintain a higher level of short-term memory for brain health, it is important that the acute increase in systemic BDNF is repeated [69]. In this connection, the effect of chronic exercise on cognitive function may be determined by repeated single exercise bout-induced physiological effects, as seen in muscle hypertrophy by resistance exercise training, and changes in some physiological and biological factors (e.g., BDNF) during single bouts of exercise may partially link such determination.

In line with this, the indirect effects of lactate should be a focus. Again, general structural alterations of the brain via chronic (i.e., repeated/habitual) exercise training/physical activity may be responsible for brain health, at least partly by growth factors. Interestingly, lactate infusion at rest induced an increase in blood BDNF in young male sports students [76]. Additionally, an increase in blood lactate concentration in response to acute graded exercise was correlated with an increase in serum BDNF in young, healthy subjects [77]. In this regard, it is not surprising that HIIE, which produces more lactate than MCE, increased serum BDNF more than MCE in young obese subjects [78]. Furthermore, acute sprint interval exercise-induced elevation in blood lactate concentration was associated with increased blood BDNF, IGF-1, and VEGF and improved cognitive performance in young subjects [79]. In addition, Hayek et al. suggested that exercise-produced lactate is transported through the circulation to the brain, whereby it induces BDNF expression via a signaling cascade between silent information regulator 1 (SIRT1), peroxisome proliferator-activated receptor gamma coactivator 1-alpha (PGC-1α), and fibronectin type III domain containing 5 (FNDC5) in the mouse hippocampus [80]. Importantly, the study also showed that such peripheral delivery of exercise-produced lactate promotes cognitive performance, such as learning and memory. These results suggest that either exercise-induced or exogenously administered lactate can be a trigger to augment BDNF expression (see [81]) and subsequent structural adaptations and hence may contribute to the improvement of cognitive performance.

Regarding VEGF, Morland et al. demonstrated that HIIE training and/or sodium lactate injections for 7 weeks promoted cerebral VEGF and angiogenesis via the lactate receptor hydroxycarboxylic acid receptor 1 (HCAR1) in an animal model [82]. These findings suggest that exercise-induced elevation of blood lactate can be an activator of neurogenesis and angiogenesis, which are favorable for brain health and should be considered an underlying molecular mechanism of HIIT benefits for the brain [83].

**Figure 3:**
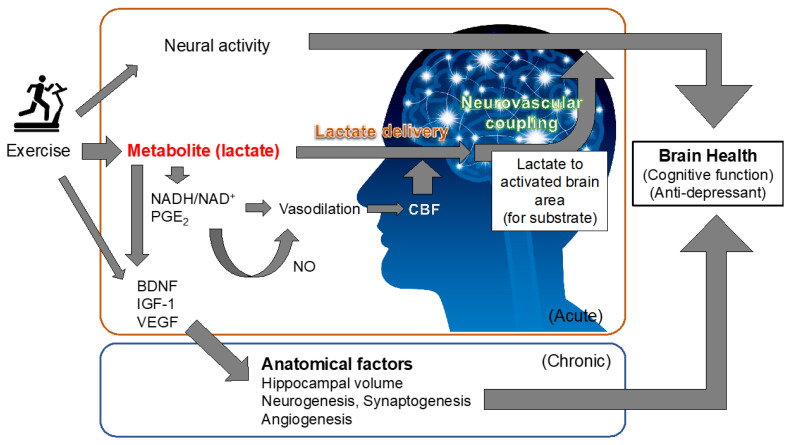
Potential acute and chronic effects of exercise-induced lactate on brain health. Scheme illustrating the potential acute and chronic effects of exercise-induced lactate on brain health.

Interestingly, previous studies demonstrated that peripheral administration of lactate reduced behavioral despair and anhedonia-like behavior and reversed social avoidance [84,85]. It was suggested that the lactate-induced expression of genes/proteins related to neuronal plasticity, memory, neurogenesis, and neuroprotection, such as BDNF, VEGF, early growth response 1 (Egr1), CCAAT/enhancer-binding protein (C/EBP), Hes5, p11, and proto-oncogene c-Fos (c-Fos), as well as activity-regulated cytoskeletal-associated protein (Arc), might be associated with the antidepressant actions of lactate [66,84,86,87]. Recently, Carrard et al. suggested that hippocampal neurogenesis is important in the antidepressant actions of lactate [84]. In this study, chronic administration of corticosterone induced depression-like states with decreased hippocampal neurogenesis, while coadministration of lactate maintained hippocampal neurogenesis to the control level with suppression of oxidative stress. Importantly, this action was not induced by the administration of pyruvate but was elicited by β-hydroxybutyrate, which can be oxidized to acetoacetate with the production of nicotinamide adenine dinucleotide hydrate (NADH), suggesting that the antidepressant effect of lactate is associated with lactate oxidation-induced NADH rather than an energy substrate [84]. Indeed, NADH suppressed corticosterone-induced oxidative stress and a subsequent reduction in adult hippocampal stem/progenitor cell proliferation in an in vitro study [84]. Although physical activity/exercise-induced physiological strain that elicits brain functional adaptation may be multifactorial [83], we should recognize that muscle contraction-produced lactate might be a pivotal mediator of brain adaptation as a myokine for brain structure (Figure 3).

## 7. Can Cerebral Blood Flow Regulation That Determines Brain Function Be Modified by Lactate?

Biochemical regulation of the cerebrovascular system by lactate is also evident in an acute setting. Gordon et al. demonstrated in rat brain slices that low oxygen levels facilitated lactate; hence, prostaglandin E_2_ (PGE_2_) elicited vasodilation [88]. In humans, the CBF response to physiological activation induced by visual stimulation was increased with lactate injection and plasma lactate/pyruvate ratio and subsequently augmented the NADH/NAD^+^ ratio [89]. This increase in lactate/pyruvate and NADH/NAD^+^ ratios may be related to the increase in CBF, probably through nitric oxide (NO) production [90]. In a clinical setting, hypertonic lactate injection increased cerebral perfusion and brain glucose availability and decreased the pulsatility index after acute brain injury [91]. In addition, the brain-injured person is hypermetabolic, and lactate has a pivotal role in supplying energy to bypass the restriction in glycolytic flux and spare limited glucose reserves for other cerebral metabolisms (e.g., pentose phosphate pathway for neuroprotection) (see [92]). Indeed, acute lactate infusion into mild traumatic brain injury patients improved their cognitive function as evaluated by the Mini Mental State Examination (MMSE), with several possible mechanisms, such as the energy substrate effect, the prevention of hyperchloremia, and the reduction in brain cell edema, by restoring impaired brain homeostasis and synapse function after brain injury [93].

## 8. Therapeutic Example of Exercise Modification to Consider the Interaction of Lactate

Given that resistance exercise is associated with several health benefits, such as a reduced risk for sarcopenia, osteoporosis, and metabolic dysfunction [94], this type of exercise is also attractive for improving quality of life. We found that an acute bout of localized resistance exercise could enhance cognitive performance immediately after exercise in a dose-dependent manner [95], whereby generally, high-intensity resistance exercise produces more lactate. Recently, we also found that resistance exercise with slow movement and tonic force generation improved EF more effectively than normal velocity movement exercise, accompanied by a considerable amount of lactate production even though the exercise intensity was low [96]. Interestingly, despite the application of a lower exercise load, resistance exercise with slow movement and tonic force generation improved postexercise EF similarly to high-intensity resistance exercise, which may be due to the equivalent blood lactate response between the two protocols in healthy young adults [97]. Therefore, it may be relevant to focus on exercise-induced lactate to predict the proper chronic exercise prescription for brain health.

## 9. Summary and Future Perspective

The potential mechanisms underlying the favorable effects of habitual exercise/physical activity on brain function are assumed to be multidimensional. In particular, structural alterations of the brain, such as increased neurogenesis, synaptogenesis, angiogenesis, and brain volume, might be characteristics of chronic exercise benefits because they cannot be achieved with only a single bout of acute exercise, although the cumulative effects of acute exercise-induced physiological stress are needed. It may be reasonable to say that acute exercise, if it is favorable for improvement of brain function, although it is a transient response, is also beneficial for brain health, including cognitive performance, with its continuous repetition via chronic exercise training. In this regard, it may be useful to understand the impact and mechanisms behind the favorable effects of acute exercise on brain function to develop a proper exercise prescription for brain health. Although such mechanisms are assumed to be multifactorial, cerebral metabolism may be an important physiological factor that determines brain function. Among metabolites, the provision of lactate to meet elevated neural activity and to regulate the cerebrovascular system and redox states in response to exercise may be responsible for exercise-enhanced brain health (Figure 3).

For this connection, the regulation of peripheral and cerebral lactate metabolism through exercise may be important for brain function. Furthermore, exercise intensity, duration, and modality also affect brain function possibly through the “metabolic myokine” (i.e., lactate). Particularly, HIIE might be practically relevant for brain health. Nonetheless, population (i.e., young and old) and gender (i.e., male and female) differences must be considered in future studies.
